# All insects are equal, but some insects are more equal than others

**DOI:** 10.1108/BFJ-05-2017-0267

**Published:** 2018-04-03

**Authors:** Arnout R.H. Fischer, L.P.A. (Bea) Steenbekkers

**Affiliations:** Wageningen University, Wageningen, The Netherlands

**Keywords:** Consumer acceptance, Entomophagy, Insect, Sub-categorisation

## Abstract

**Purpose:**

Lack of acceptance of insects as food is considered a barrier against societal adoption of the potentially valuable contribution of insects to human foods. An underlying barrier may be that insects are lumped together as one group, while consumers typically try specific insects. The purpose of this paper is to investigate the ways in which Dutch consumers, with and without insect tasting experience, are more or less willing to eat different insects.

**Design/methodology/approach:**

In a quasi-experimental study (*n*=140), the participants with and without prior experience in eating insects were asked to give their willingness to eat a range of insects, and their attitudes and disgust towards eating insects.

**Findings:**

Insects promoted in the market were more preferred than the less marketed insects, and a subgroup of preferred insects for participants with experience in eating insects was formed.

**Research limitations/implications:**

Although well-known insects were more preferred, general willingness to eat remained low for all participants. The results indicate that in future research on insects as food the specific insects used should be taken into account.

**Practical implications:**

Continued promotion of specific, carefully targeted, insects may not lead to short-term uptake of insects as food, but may contribute to willingness to eat insects as human food in the long term.

**Originality/value:**

The paper shows substantial differences between consumers who have and who have not previously tasted insects, with higher acceptance of people with experience in tasting insects for the specific insects that are frequently promoted beyond their generally more positive attitude towards eating insects.

## Introduction

In the next few decades, the growing world population and increasing societal welfare will double the demand for high-quality proteins for human consumption; while at the same time, the ecological footprint of the current food production needs to be halved to ensure long-term sustainability. As a consequence, traditional livestock production needs to be supplemented as a main source of protein for human consumption by other sources of protein (Boland *et al.*, 2013). Insects typically contain from 30 to 81 per cent protein per 100 grams dry-weight; which is comparable to protein contents of meats such as beef or pork. Insects are also rich in essential amino acids and unsaturated fatty acids. Some insects contain important (trace) minerals and vitamins in quantities higher than in most animal or plant food products (Ramos-Elorduy, 2009). For example, termites are rich in calcium and sulphur whereas grasshoppers are rich in iron and zinc (Ramos-Elorduy, 2009). Insects are also generally rich in niacin, riboflavin and thiamine (Rumpold and Schlüter, 2013). Insects require less energy and space to grow than meat-producing animals (Van Huis *et al.*, 2013). Food conversion rates of insects are better than even efficient meat sources like poultry (Rumpold and Schlüter, 2013). Food waste can, in some situations, be used to breed insects, although this is not yet allowed in the EU (Finke *et al.*, 2015). There are close to 2,000 edible insects which include beetles, butterflies, moths, wasps, bees and ants (Ramos-Elorduy, 2009). In addition, insects are eaten in various stages of maturity; for example mealworms, the larvae stage of the mealworm beetle, are consumed, while mature crickets and locusts are eaten (Zielińska *et al.*, 2015). Together these properties make insects a potential sustainable supplement to the existing protein sources for human nutrition.

However, for this potential to be reached the insects and/or insect products need to be consumed. Entomophagy or purposely eating insects is found in about 130 countries, but is not common in European and American industrialised cultures (Van Huis *et al.*, 2013). In those cultures, insects as a food source have long been limited to the menu of certain niche restaurants and to small-scale companies and retailers selling packaged, often freeze dried, insects for consumption. More recently, insect tastings (Shelomi, 2015) and some limited sales in supermarkets have aimed at larger scale introductions of insects as food in European countries such as the Netherlands (House, 2016). In particular mealworms, crickets and grasshoppers were introduced to the Dutch consumers through tastings and web-shops[1] and via insect cookbooks (e.g. Van Huis *et al.*, 2014).

Nevertheless, published studies in Europe show low acceptance across the continent. Members of the Dutch public have a low expected liking of insect foods (Tan, Fischer, Van Trijp and Stieger, 2016), an aversion to eating insects and a low acceptance of insects as food source (Schösler *et al.*, 2012, Looy *et al.*, 2014). The aversion to eating insects is differentiated across the population, with some segments of the Belgian population in particular those showing less neophobia being more open to the consumption of insects than others (Verbeke, 2015), and German consumers with a higher level of neophobia being less willing to eat insects (Hartmann *et al.*, 2015), suggesting neophobia as a relevant predictor to acceptance of insect eating.

Much of the research has been done on the appropriateness of the product in which insects are invisibly used both in European (Tan *et al.*, 2017; Gmuer *et al.*, 2016) but also in African countries (Alemu *et al.*, 2017). Retail introductions of insect burgers have followed such a product-centred approach by introducing insect burgers containing invisible insects (House, 2016). This is based on the finding that disgust is lower when insect protein is applied as invisible, ground ingredient (Caparros Megido *et al.*, 2016). A study using crickets among Swiss participants showed that, at least in that case, it was the inappropriate combination of visible crickets with known food products that resulted in very negative responses, even more strongly so than the crickets presented as standalone food (Gmuer *et al.*, 2016). While the approach of investigating insect acceptance as food in general, or focussing on specific insects certainly has value, it may lead to overgeneralisation of conclusions if consumers have systematically different evaluations of different insects. The current paper aims to provide insight into how different insects evoke different levels of willingness to eat these insects by consumers.

Disgust (Looy *et al.*, 2014), considerations of insects as being inappropriate for human consumption (Tan, Fischer, Van Trijp and Stieger, 2016) or inappropriately being added to the existing foods (Gmuer *et al.*, 2016; de-Magistris *et al.*, 2015) are raised as barriers to the willingness to eat insects. Some studies suggest that these barriers depend on inborn disgust and that this creates a lasting negative affective attitude towards eating insects in general and that only cultural reinforcement can make eating insects accepted (Ruby *et al.*, 2015). Other research does argue that the disgust evoked towards insect eating may be the learned cultural response (Deroy *et al.*, 2015). Regardless of whether disgust is inborn or learnt, a distinction seems to be made between more and less accepted insects, which can be found among populations in Africa that consume specific insects, while neighbouring populations (with access to the same wildlife) do not consume those same insects (Van Huis, 2003). People from a Thai region in which entomophagy is common, most appreciated insects that were food ingredients in their cuisine and occurred locally, but were disgusted by the mealworms which they associated with spoilage (Tan *et al.*, 2015). While these studies focus on the cultures where insect eating is fairly common, a study in a related field shows that the Dutch people do take due account of the species of animals when deciding whether a product is acceptable to eat (Bekker, Tobi and Fischer, 2017), and results from Tan *et al.* (2015) show that the preference order of insects among the Thai people differed from that of the Dutch with prior experience in insect tasting. Thai people were in fact more disgusted by mealworms than the Dutch people with prior experience. In a comparative study between German and Chinese consumers, it was found that Chinese consumers do distinguish between crickets and culturally known silkworms, while Germans do not (Hartmann *et al.*, 2015). Other research showed that among Czech consumers, some insects (cockroaches) evoke more disgust than others (crickets) (Bednárová *et al.*, 2013). This suggests that even in countries were insect eating is not common, consumers distinguish between different insects, but that experience with specific insects matter in the extent consumers are willing to accept these specific insects as food.

In spite of these different practices, much of the current debate about entomophagy is about insect eating in general (Evans *et al.*, 2015), i.e. focussing on a single overarching category of insects. From a consumer point of view, categorisation is a way to make sense of a product in the given context by placing it in a category that provides maximal fit with other category members (Mervis and Rosch, 1981). While it makes sense to place insects in a global category if we merely see them as animals, if consumers consider insects in the perspective of food, a global taxonomic insect category may not suffice, and a different, goal based category is more relevant (cf. Huffman and Houston, 1993). In such cases where global categories do not fit a use context consumers tend to create subcategories to interpret specific products from the global category in the goal context (cf. Sujan and Dekleva, 1987; Ratneshwar and Shocker, 1991). This leads to different inferences of product properties (Redden, 2008) which in the case of insects can lead to a distinctly different appreciation of the subcategory of food insects. The creation of subcategories requires some experience in order to reconsider specific products as member of a specific subcategory (Johnson and Mervis, 1997; Tanaka and Taylor, 1991). In the case of insects, we would therefore expect that a particular group of insects that is more consistently shown as food in specific recipes (e.g. as in Van Huis *et al.*, 2014), and that is presented as food to and tasted by the public would be subcategorised as food insects, and in particular by those consumers that have at least some experience in eating them.

Therefore, we hypothesise that for people with no prior experience in eating insects, preferences towards eating specific insects will follow more or less a continuum from very unfavourable to unfavourable, while for those who do have experience in eating insects, the insects most frequently available as food in the region inhabited by the consumer will stand out as a more distinct and more preferred subcategory.

## Method

The study included a two level (no prior experience-prior experience with eating insects) quasi-experimental factor. In addition, 17 different insects were rated on willingness to eat by each participant. To test the hypothesis that insects known as food would be subcategorised as food insects, the three insects most generally presented to the public in tastings, insects festivals and retail in the Netherlands (mealworm, cricket, grasshopper)[2] were included. To test a broad view across edible insects, these were presented as part of a set of 17 insects consisting of more broadly liked insects (grasshopper, butterfly, dragonfly, caterpillar) more neutral or ambivalently regarded insects (cricket, beetle, moth, bee) infrequently mentioned insects (termite, worm, water bug) and generally disliked insects (cockroach, ant, wasp) (Shipley and Bixler, 2017). In addition, we included insect eggs, and included a subfamily of grasshopper: slantface (*Acridinae*) to include a largely unknown insect name as control.

Participants were invited through leaflets and internet forums or social media to participate in a study on insect eating. The participants were students at the Wageningen University. Wageningen has hosted several insect tasting trials, and entomophagy is taught and studied in several departments at the Wageningen University, which makes it more likely to identify the participants who had experience in eating insects than when using the general population. Sampling included an active search for the participants with experience in eating insects. Data were collected in 2014. The final data set consisted of 140 surveys[3]. Of the participants, 54 per cent (76) were female, 36 per cent male (50) and 10 per cent (14) did not report their gender. Average age was 24.9 years (SD=7.2). Slightly more than half (*n*=77) had not eaten insects and 45 per cent (*n*=63) had (deliberately) eaten insects.

### Measures

Willingness to eat an individual insect was measured on a single five-point item “To what extent would you be willing to eat (named insect)” ranging from “1: very unlikely” to “5: very likely”. In addition, general attitude towards eating insects was measured with a reliable 19-item, seven-point semantic differential attitude scale (adapted from Crites *et al.*, 1994) containing a general attitude towards eating insects subscale (four items, Cronbach’s *α*=0.93) an affective attitude subscale (seven items, Cronbach’s *α*=0.94) and a cognitive attitude subscale (eight items, Cronbach’s *α*=0.88) (previously used in a similar context by Bekker, Fischer, Tobi and Van Trijp, 2017). In addition, the general acceptance of insects as food was measured with a nine-item seven-point Likert scale (Cronbach’s *α*=0.90) (adapted from Schutz, 1965). General disgust towards eating insects was measured with a six-item seven-point Likert scale (Cronbach’s *α*=0.91) (adapted from Rozin and Fallon, 1980). Food neophobia was also measured using an eight-item seven-point Likert scale (Cronbach’s *α*=0.81) (adapted from Pliner and Hobden, 1992). An overview of all items is provided in the Appendix. As all Cronbach *α*s were above the recommended limit of 0.70 (Nunnally, 1978), the scales were deemed reliable and average item scores were calculated to represent the measured constructs. After a pre-test with ten participants to determine whether questions were understandable, no adjustments were needed.

### Procedure

The participants were mailed a link and could complete the questionnaire from any internet connection at their own timing. When following the link they were first introduced to the research. They then received the information that “Insects are eaten in 130 countries in the world, there are over 2,000 species of insects that can be eaten”.

The participants then answered whether they had ever (deliberately) eaten insects themselves, followed by rating their attitude towards eating insects in general. They then filled out general acceptance of insects as foods, disgust and neophobia items, following by their willingness to eat the individual insects. The study ended with questions about age, gender and study programmes, after which the participants were thanked for their involvement in the study.

## Results

Of those who had tried insects before (*n*=63), 68 per cent (43) reported they would be willing to eat them again, 5 per cent (three) would not be willing to eat them again and 27 per cent (17) were not yet sure. Almost all participants with experience in eating insects (98 per cent) reported their insect consumption was rare and was at most a few times per year.

Disgust, general, affective and cognitive attitude towards and acceptance of insects as food and neophobia were all significantly correlated in the expected direction (Table I). A logistic regression showed that the likelihood of having experience with eating insects depends on neophobia (*χ*^2^(1)=5.02; *p*=0.03; Nagelkerke *R*^2^=0.05, odds ratio: 1.51), to the extent that people with higher neophobia less frequently had experience with eating insects.

**Figure 1:**
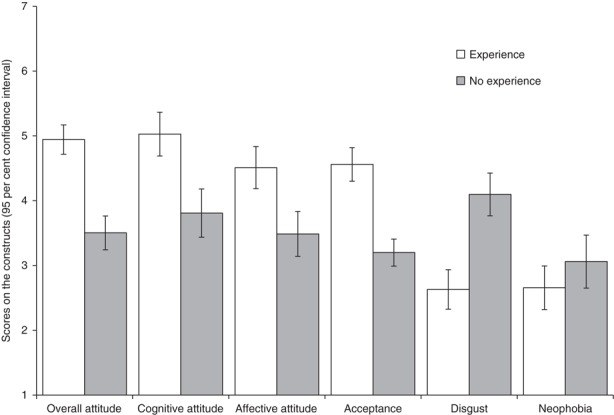
Means and 95 per cent confidence interval (measured on seven-point scales) for the constructs related to response towards eating insects in general for those with and without prior experience in eating insects

A multivariate analysis of variance showed that the participants with experience in eating insects were significantly more positive in overall attitude (*F*(1,130)=31.55, *p*<0.001, *η*^2^=0.20), cognitive attitude (*F*(1,133)=28.90, *p*<0.001, *η*^2^=0.18), affective attitude (*F*(1,138)=48.14, *p*<0.001, *η*^2^=0.26) and acceptance of insects as foods (*F*(1,127)=34.71, *p*<0.001, partial *η*^2^=0.21). Insect-eating people scored lower on disgust (*F*(1,127)=28.31, *p*<0.001, partial *η*^2^=0.18) (Figure 1).

**Figure 2:**
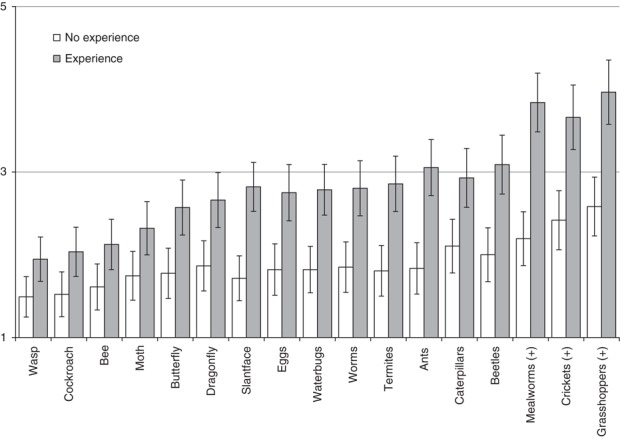
Mean (95 per cent confidence interval) of willingness to eat (1=very unlikely; 5=very likely) specific insects for participants who had, and had not prior experience with eating insects. Insects marked (+) were available for human consumption in the Netherlands at the time of data collection

A repeated measures ANOVA was conducted with the willingness to eat each of the 17 insects as the repeated factor and the quasi-experimental factor whether a participant had experience with eating insects. The analysis confirmed that the participants with experience with eating insects scored on average more positive towards the insects (*F*(1,121)=27.04, *p*<0.001, partial *η*^2^=0.18). More importantly the repeated measures analysis revealed that not all insects were liked the same (*F*(1,121)=36.19, *p*<0.001, partial *η*^2^=0.23 (lower bound)); and, in line with our hypothesis the interaction between the repeated factor (insect) and whether participants had experience with eating was significant (*F*(1,121)=5.25, *p*=0.02, partial *η*^2^=0.04). Simple effects analysis showed that the differences between insects were significant for both those with experience (*F*(16,106)=10.04, *p*<0.01, partial *η*^2^=0.60,range of 2.01 scale point) and for those without experience with eating insects (*F*(16,106)=2.90, *p*<0.01, partial *η*^2^=0.31, range of 1.09 scale point) (Figure 2).

After establishing that the participants with experience in eating insects more clearly distinguished between insects in their willingness to eat insects than those who have no experience, we focused on the individual insects (Table II). Pairwise comparisons between specific insects were made (Bonferroni corrections for multiple comparisons). The participants with experience in eating insects showed a distinct subcategory of most favoured insects, being those insects most frequently used in tastings (mealworms, crickets and grasshoppers). In addition, a substantial number of insects not readily available for consumption (dragonfly, eggs, water bugs, worms, termites, ants, caterpillars and beetles) were both significantly and relevantly favoured (as evidenced by partial *η*^2^ above 0.09) by the participants with experience with eating insects over participants without experience. For the more negatively rated butterfly, moth, bee, wasp and cockroach participants with experience with eating insects were significantly more willing to eat those compared to participants without experience, but these differences were small (as evidenced by partial *η*^2^ between 0.01 and 0.09); indicating that the difference between eaters and non-eaters was less pronounced for these insects.

Using a general linear model, with the 17 insects as repeated measure and overall attitude, affective attitude, cognitive attitude, general acceptance of insects as food and neophobia as predictor, showed similar regression coefficients for each predictor regardless of the specific insect (all *F*s<1.6, all *p*s⩾0.10). A subsequent multiple regression analysis on the aggregated willingness to eat across all 17 insects only showed effects of disgust and affective attitude on willingness to eat insects (Table III). Adding the interaction effects of those who had previously consumed insects or not with the predictors was not statistically significant (*F*_change_(6,112)=0.60, *p*=0.73). The noticeable lack of effect of neophobia suggests that the effect of neophobia is mediated through attitude components rather than being a proximal effect on willingness to eat insects.

Some caution is warranted in interpreting the analyses reported in Table III, as collinearity statistics for the different attitude components, including overall acceptance of insects as food, are only just acceptable. What can be concluded is that emotional responses (affective attitude component and disgust) are the strongest predictors of willingness to eat insects, and that in this case where attitudes and disgust are included neophobia has limited to no additional explanatory value for willingness to eat insects.

## Conclusions and discussion

This study confirms and combines findings from the previous studies on European samples by showing that those with experience in eating insects before show a higher willingness to eat insects again (as shown by Tan, Van Den Berg and Stieger, 2016), and that there are significant differences in willingness to eat different insects (as shown by Zielińska *et al.*, 2015). This suggests that disgust and attitude towards the idea of eating insects in general is not the only factor predicting the willingness to eat specific insects. The four insects the participants were least willing to eat; wasp, cockroach, bee and moth were listed elsewhere as disliked (cockroach, wasp), or ambivalent (bee, moth) (Shipley and Bixler, 2017). The insects (grasshoppers, crickets and mealworms), most often marketed as food in the culture of the participants, were those they are most willing to eat. These results align with the results by an earlier study in a Thai insect-eating culture (Tan *et al.*, 2015) where the participants were not willing to eat insects not readily available for consumption within that culture, even when these insects are consumed in other cultures. Our findings thus suggest that trying specific insects influences the willingness to eat those specific insects more than the willingness to eat an insect in general. A further indication for this subcategorization can be found in the observation that the insects most frequently marketed as food (cricket, grasshopper, mealworm) formed a significantly distinct group of insects with the highest willingness to eat, that stood apart from the other insects for participants with experience in eating insects, but not for those without such experience. Similar sub-categorisation may also influence the introduction of other new foods such as algae or meat replacers.

General disgust and the general affective attitude component most frequently had a (negative) effect on willingness to eat a specific insect. The finding that disgust is an important predictor of (non)willingness to eat insects replicates the existing research (e.g. Looy *et al.*, 2014). Where Gmuer *et al.* (2016) similarly show that more emotions than only disgust matter in willingness to eat insects, the current research adds to that, that affective attitude contributes to willingness to eat beyond more cognition-based evaluations. Thus, our results further emphasise that willingness to eat insects is driven to a large extent by emotions.

The finding in the current study that neophobia did not significantly contribute to willingness to eat insects seems at odds with other research in willingness to eat insects that found significant and substantial contributions of neophobia to willingness to eat insects (Hartmann *et al.*, 2015; Verbeke, 2015). In other studies, much smaller effects of neophobia where found however (Tan, Fischer, Van Trijp and Stieger, 2016; Tan, Van Den Berg and Stieger, 2016). If we compare these studies, it is noticeable that the studies of Hartmann and colleagues and Verbeke do not include any evaluation of eating insects as predictor of willingness to eat insects, while the other studies included evaluations of insect products besides neophobia, i.e. of perceived appropriateness (Tan, Fischer, Van Trijp and Stieger, 2016) and anticipated taste (Tan, Van Den Berg and Stieger, 2016). The current study includes a measure of evaluation (i.e. attitude as defined in e.g. Eagly and Chaiken, 1993) of insects as food. Therefore, we argue that neophobia is not a proximal determinant of willingness to eat insects but rather a further removed determinant that exerts its influence through perceptions and evaluations. This makes sense if we consider that neophobia is a personality trait (Pliner and Hobden, 1992) and therefore likely to be an antecedent for all perceptions and evaluations in the mind. Future research should focus on how to model neophobia in more complex causal models both within the context of eating insects, but also for other new foods.

A practical implication and limitation is that although there were clear differences between those with and without experience with eating insects, willingness to eat insects was fairly limited even among those who had previously eaten insects. In addition, we should take into account that reported willingness to eat tends to be higher than the acceptance of insects as part of the regular diet (Tan, Fischer, Van Trijp and Stieger, 2016). Therefore, the willingness to include insects in the regular diet is probably even lower than the estimates in this study. The sample consisted of volunteers who were invited to a study on insect eating. In addition, the sample mainly consisted of the students of Wageningen University, which has been one of the centres of entomophagy in Europe which may have favoured their opinion about eating insects. This may have somewhat biased the sample towards the participants more than averagely positive about the idea of insects eating, and this would likely increase differences between those who had experience with eating insects (and thus shown a somewhat positive attitude towards insects eating) and those who had not. Future research should investigate to what extent the current results can be generalised. In combination with other, practical barriers identified in other research, such as the lack of understanding how to prepare and what to expect of the taste of whole insects (Tan *et al.*, 2015), and limited availability of insect products (House, 2016; Shelomi, 2015), the shown low willingness to eat insects even for those who had eaten insects before shows that acceptance of insects as food remains problematic.

The current results do suggest that future effort to promote insects as food should include a systematic approach to identifying which insects are suitable for continued introduction in Europe. The current study shows that the introduction of specific insects as food may have resulted in the reduction of negative sentiment to eating at least some insects, as particularly people who already reported experience with eating insects showed distinctly higher willingness to eat these insects. This success remains limited at best, however, as willingness to eat even those insects was fairly low. Nevertheless, continued investment in promoting specific insects besides overcoming other practical barriers identified in other research may contribute to higher willingness to eat insects in the future.

For now, we would suggest that at least in the Netherlands, and at this moment in time promotion efforts for grasshoppers, mealworms and crickets – the insects highest scoring on willingness to eat – may be the best way forward in the short term.

## Figures and Tables

**Table I:** Pearson correlations between attitude, acceptance, disgust and neophobia

	Cognitive attitude	Affective attitude	Acceptance	Disgust	Neophobia
Overall attitude	0.87**	0.83**	0.77**	−0.73**	−0.41**
Cognitive attitude		0.78**	0.67**	−0.64**	−0.40**
Affective attitude			0.76**	−0.70**	−0.41**
Acceptance				−0.79**	−0.43**
Disgust					0.42**

**Notes:** *p<0.05; **p<0.01

**Table II:** Comparison between willingness to eat (on a scale from 1 – very unlikely, to 5 – very likely) specific insects between participants with and without experience in eating insects

	Experience (*n*=56)	No Experience (*n*=67)	Comparison between experienced and non-experienced eaters		
	Mean (95% CI)	Mean (95% CI)	Mean difference	Significance	Partial *η*^2^
Wasp	1.95 (1.68, 2.21)^a^	1.49 (1.25, 1.74)^a^	0.45	0.014	0.05
Cockroach	2.04 (1.74, 2.33)^ab^	1.52 (1.25, 1.79)^ab^	0.51	0.013	0.05
Bee	2.13 (1.82, 2.43)^abc^	1.61 (1.33, 1.89)^abc^	0.51	0.015	0.05
Moth	2.32 (2.00, 2.64)^abcd^	1.75 (1.45, 2.04)^abc^	0.58	0.010	0.05
Butterfly	2.57 (2.24, 2.90)^bcde^	1.78 (1.47, 2.08)^abc^	0.80	0.001	0.09
Dragonfly	2.66 (2.33, 2.99)^cde^	1.87 (1.56, 2.17)^abc^	0.80	<0.001	0.20
Slantface	2.82 (2.53, 3.12)^e^	1.72 (1.45, 1.99)^abc^	1.11	0.001	0.09
Eggs	2.75 (2.41, 3.09)^de^	1.82 (1.51, 2.13)^abc^	0.93	<0.001	0.12
Water bugs	2.79 (2.48, 3.09)^de^	1.82 (1.54, 2.10)^abc^	0.96	<0.001	0.15
Worms	2.80 (2.47, 3.14)^de^	1.85 (1.55, 2.15)^abc^	0.95	<0.001	0.15
Termites	2.86 (2.52, 3.19)^e^	1.81 (1.50, 2.11)^abc^	1.05	<0.001	0.13
Ants	3.05 (2.72, 3.39)^e^	1.84 (1.53, 2.15)^abc^	1.22	<0.001	0.19
Caterpillars	2.93 (2.57, 3.28)^e^	2.10 (1.78, 2.43)^cd^	0.82	<0.001	0.09
Beetles	3.09 (2.73, 3.44)^e^	2.00 (1.67, 2.33)^bcd^	1.09	<0.001	0.14
Mealworms (+)	3.84 (3.48, 4.19)^f^	2.19 (1.87, 2.52)^cde^	1.65	<0.001	0.28
Crickets (+)	3.66 (3.27, 4.05)^f^	2.42 (2.06, 2.77)^de^	1.24	<0.001	0.15
Grasshoppers (+)	3.96 (3.58, 4.35)^f^	2.58 (2.23, 2.94)^e^	1.38	<0.001	0.18
Average across all insects	2.84 (2.57, 3.10)	1.89 (1.65, 2.13)	0.95	<0.001	0.18

**Notes:** Cells within the same column sharing a superscript character are not significantly different at *p*=0.05 (Bonferroni correction). Insects names marked (+) were available for human consumption in the Netherlands at the time of data collection

**Table III:** Regression weights for multiple regressions of determinants of the average willingness to eat specific insects (on a scale from 1 – very unlikely, to 5 – very likely)

		95% CI					
	Regression coefficient	Lower bound	Upper bound	Standardised regression coefficient	Tolerance (collinearity statistic)^a^	*t* (118)	*p*
Constant	1.49	0.42	2.57				
Overall attitude	0.13	−0.07	0.32	0.19	0.10	1.24	0.22
Cognitive attitude	−0.05	−0.21	0.11	−0.06	0.20	−0.58	0.56
Affective attitude	0.26	0.04	0.48	0.24	0.22	2.35	0.02
Acceptance of insects as food in general	0.12	−0.06	0.31	0.19	0.15	1.31	0.19
Disgust with eating insects	−0.25	−0.36	−0.15	−0.39	0.35	−4.93	<0.001
Food neophobia	−0.019	−0.17	0.13	−0.01	0.74	−0.25	0.81
Experience with eating insects (−0.5: no, +0.5: yes)	0.04	−0.21	0.29	0.02	0.72	0.32	0.75

**Notes:** ^a^Tolerance does not surpass the critical limit of 0.10 and overall attitude is therefore maintained; Model 2 – not reported shows no significant contribution of two-way interaction terms between previous insects’ consumers and the other predictors. Overall model: *F*(7,118)=47.08, *p*<0.01; *R*^2^=0.74. Model 2: *F*(13,112)=25.12, *p*<0.01; *R*^2^=0.74; *F*-test for significant increase in *R*^2^ (*H*_0_: no change): *F*_change_ (6,112)=0.60, *p*_change_=0.73

## Notes

1. www.delibugs.nl/Webwinkel-Category-3794795/Eetbare%20insecten.html (accessed 3 May 2017); available at: www.webpoelier.nl/bugs.html (accessed 3 May 2017).

2. http://duurzaaminsecteneten.nl/insecten-kopen/ (accessed 30 August 2017).

3. In total, 17 incomplete surveys were encountered which were used for analyses if applicable resulting in some variations in *n* and dfs in the results section.

## Appendix: Scale items

All items measured on seven-point scales. (R) indicates recoded items. English version of the used items. The Dutch version used in the research is available from the first author on request.

Adapted from Crites *et al.* (1994), “When I think I have to eat insects I feel:” Affect: hateful-love; sad-delighted; annoyed-happy; tense-calm; bored-excited; angry-relaxed; disgusted-acceptance; sorrow-joy. Cognition: useless-useful; foolish-wise; unsafe-safe; harmful-beneficial; worthless-valuable; imperfect-perfect; unhealthy-healthy. General: negative-positive; dislike-like; bad-good; undesirable-desirable.

Adapted from Schutz (1965), general acceptance towards eating insects (disagree completely-agree completely): “I would eat insects every opportunity I have”; “I would eat insects if available but would not go out of my way”; “I would eat insects only if there were no other food choices”(R); “I would eat insects in the future”; “I like insects and would eat them now and then”; “ I don’t like the taste, smell or texture of insects” (R); “I do not like the appearance of insects” (R); “I will never eat insects” (R).

Adapted from Rozin and Fallon (1980), the disgust towards eating insects (disagree completely-agree completely): “The thought of eating insects makes me nauseous”; ”I dislike the idea of having insects in my stomach”; “I would dislike it if insect food got on my hands”; “I would dislike any dish that contained even the tiniest amount of insects”; “I dislike insect food because of the idea what it is or where it comes from”; “I feel that insect food might contain something that might physically endanger my body”.

Adapted from Pliner and Hobden (1992), food neophobia (disagree completely-agree completely): “I am constantly sampling new and different foods” (R); “I don’t trust new foods”; “I like foods from different countries” (R); “ethnic food looks too weird to eat”; “at dinner parties, I will try a new food” (R); “I am very particular about the foods I will eat”; “I will eat almost anything” (R); “ I like to try new ethnic restaurants” (R).
